# Why do first-time mothers not intend to breastfeed? ——A qualitative exploratory study on the decision-making of non-initiation in Jingzhou, China

**DOI:** 10.1186/s12884-022-04494-5

**Published:** 2022-03-07

**Authors:** Yang Fei, Ze-Yu Zhang, Wen-Ning Fu, Li Wang, Jing Mao

**Affiliations:** 1 School of nursing, Tongji Medical College, Huazhong University of Science and Technology, Hangkong Road 13, Wuhan, China; 2 Yangtze University Medical School, Nanhuan Road 1, Jingzhou, China; 3 Jingzhou First Hospital, Hangkong Road, JingZhou, China

**Keywords:** Breastfeeding, Non-initiation, Qualitative study

## Abstract

**Background:**

Although breastfeeding is one of the top priorities for public health in China, the rate of breastfeeding is still low and a large number of women do not initiate breastfeeding due to various reasons. Hence, this study aimed to understand the decision-making of non-initiation and reveal the underlying reasons in order to protect, support, and promote breastfeeding.

**Methods:**

In-depth, exploratory interviews were carried out with 13 new mothers who did not initiate breastfeeding. The collected data were analyzed by inductive content analysis.

**Results:**

Although mothers generally understood the protective effects of breastfeeding, they believed that formula milk is a good alternative to human milk and even better in some aspects. Five core themes related to non-initiation decision-making emerged: (1) expected breastfeeding stress; (2) maladjustment to the maternal role; (3) concerns about physiological issues; (4) the lack of knowledge about the risks of artificial feeding; (5) belief that it is better not to initiate than to interrupt.

**Conclusions:**

The environment for mothers to breastfeed in China is not supportive enough, and the medical staff and families should be held responsible for the non-initiation of breastfeeding. More professionals are needed to support mothers to solve their problems and breastfeeding education should be further expanded.

## Background

Breastfeeding is the most effective preventive intervention to improve maternal and children health globally. It is estimated that the deaths of 595,379 children (6 to 59 months) from diarrhea and pneumonia, and 98 243 women from breast cancer, ovarian cancer, and type II diabetes each year can be prevented if optimal breastfeeding practices are adopted [1]. Consequently, breastfeeding is recommended by the World Health Organization (WHO) [2] due to its protective effects for both mothers and children.

Despite this recommendation [2], the breastfeeding rate worldwide is not optimistic over the past few years. First time mothers no longer consider breastfeeding as the unique decision and even stop breastfeeding early or reject to initiate. Although breastfeeding is one of the top priorities for public health in China, the rates of breastfeeding are still low [3]. Indeed, China is the largest infant (0-12 month) formula market in the world, with a projected annual growth rate of 20% [4]. According to a survey [5] in 2019, the exclusive breastfeeding rate of Chinese infants under 6 months is only 29%, which is much lower than 43% of the world average and 37% of the average of low-and middle-income countries.

Determinants of breastfeeding practices are complex and operate at social, structural, and individual levels [6]. In a review of the literature on developed countries, the key positive factors include older maternal age, higher level of education, higher socioeconomic status, and healthy maternal habits [7]. Previous studies focused mainly on the early interruption of breastfeeding. According to Morrison’s study [8], the two most common reasons for early cessation were perceived inadequate milk supply and maternal breast or nipple pain. Other studies [9, 10] confirmed that obesity and smoking are risk factors. Social support characteristics (e.g., lack of breastfeeding support in the hospital, poor breastfeeding environment [11], and insufficient maternity leave [12]) have also been described as important reasons for early weaning.

Although much is known about early interruption, the low rate of breastfeeding is not entirely due to early interruption. Not all mothers are willing to breastfeed their newborns and initiate breastfeeding after giving birth. In earlier publications [13, 14], the overall prevalence of breastfeeding initiation was estimated to be 81.1% in the US, 74.0% in the UK [15], and 71.3–99.9%[16] in various cities in China. Although many postpartum mothers do not initiate breastfeeding, the research on non-initiation decision-making is very limited. According to a quantitative study [17] of Ogbuanu et al., mothers provided individual reasons and household responsibilities as reasons for not initiating breastfeeding. Hall [18]et al. reported that artificial feeding was endorsed by friends and their mothers or considered the easiest way of feeding, or no other way was considered. Horwood [19] et al. reported that the health status of mothers was the main reason for artificial feeding.

Understanding the factors related to the refusal of mothers to initiate breastfeeding is the basis for the development of an intervention program. However, the exclusive use of quantitative methodologies for studying such a complex issue oversimplifies and misses some key nuances of the breastfeeding decision-making process [20]. Previous research did not comprehensively reveal the real reasons and psychological experience leading to the decision to not initiate breastfeeding. Relevant research has not been carried out in China, and a further qualitative exploration is needed. In view of the low breastfeeding rate in China, the present study aims to understand the decision-making of non-initiation and to reveal the underlying reasons in order to promote breastfeeding.

## Methods

To obtain a comprehensive understanding of the research problems, a qualitative exploratory research design was adopted. It is the best when the researcher is seeking to better understand an experience of life that is currently not well understood or is under-researched [21].

The study was conducted in Jingzhou, a medium-sized city in Hubei Province (China) with dense population. The number of births in Jingzhou reaches 8000–10,000 per year. In total, there are five grade-3 hospitals in Jingzhou City that provide prenatal and postnatal nursing services for mothers. The hospitals have always supported breastfeeding and followed Ten steps to successful breastfeeding. A statement of rejecting breastfeeding should be required if the woman has a demand for artificial feeding after giving birth to a child, which will help researchers in accurately targeting the objects of not initiating breastfeeding. According to the Chinese traditional customs, the postpartum women are taken care by their families after hospital discharge and routinely adhere to the traditional maternal postpartum practices called “doing the month” in Jingzhou during the first month, which are believed to restore the body harmony and health and prevent future illness.

The participants were determined through purposeful sampling and criterion sampling [22]. As compared to the pregnant mothers, the non-initiation of breastfeeding is already an actual behavior for the first-time mothers rather than an intention. Therefore, the present study focused on mothers. In addition, it was found that non-first-time mothers were influenced by their past breastfeeding experiences [23]. Therefore, first-time and non-initiation mothers were recruited consecutively. The criteria for inclusion in this study were as follows: (1) full-term childbirth between 37 and 42 gestational weeks; (2) three to seven days after giving childbirth (to avoid memory bias); and (3) baby on artificial feeding regimen after birth. Mothers were excluded from the study if they had any reported mental health problems or any postpartum complications. A total of 15 pregnant mothers were invited and 2 refused, 13 eligible volunteers were interviewed at last. After the 11th interview, no new information was identified. Two more participants were interviewed to confirm that data saturation was reached. The sample size of the participants was determined by data saturation [24].

Data were collected between August and December in 2020 through semi-structured [25], in-depth interviews, and an interview guide with open-ended questions was developed to explore the psychological experience of the mothers. The first author, who was a faculty member of nursing school with a breastfeeding experience more than two years, conducted all of the interviews. The purpose of the interview was clearly explained, and the mothers who consented were interviewed in their own maternity ward. If the maternity ward was not a single room, any other spare room in the ward was used instead. Before the formal interview, the participants were informed of the principle of confidentiality and the necessity of recording, and the interviewer informed the participants that their privacy was protected by using an identification number (N1–N13) instead of their names. Demographic information was collected before the interview, and the following core questions were asked from all the mothers. (1) How did you decide to non-initiating breastfeeding? (2) Who influenced your decision? What role did these persons play? (3) What do you think of breastfeeding (advantages and disadvantages)? (4) What do you think of artificial feeding (advantages and disadvantages)? The duration of the interview was 35–70 min.

Audio recordings were stored on an encrypted, password-protected laptop and transcribed using intelligent verbatim. Transcription quality was double-checked by the first author and the third author to ensure accuracy. Audio recordings were deleted once proofreading was finalized.

The data analysis was completed by two researchers. Demographic data were analyzed using descriptive statistics. Qualitative data was analyzed using the inductive content analysis [26], which is usually applied when existing theory or research literature on a phenomenon is limited [27]. Nvivo 12 software was used and the data were analyzed as follows: All the interview transcripts were read several times to permit the researchers to immerse themselves in the data. The transcripts were coded line by line to annotate important ideas and concepts, similar and related codes were classified into categories, the categories were defined and further abstracted into larger core themes, and the examples of corresponding excerpts from the data were searched.

Four criteria, proposed by Lincoln and Guba, were used to ensure the rigor of the results, including credibility, dependability, transferability, and confirmability [28]. In order to ensure credibility, the in-depth interviews were conducted in a quiet place to focus on participants’ statements and help them feel comfortable and relaxed. By member checks, the initial coding of interviews was reviewed by the interviewees to confirm the accuracy of the codes, and the results of the study were given to three first-time mothers to judge the similarity of the results with their own experiences for increasing the transferability. In order to enhance dependability, the peer-check technique was used. Two researchers performed the coding and categorization independently, and in the case of any disagreement, to reach the consensus, discussions were made to clarify the results.

## Results

Table 1 summarizes the demographic characteristics of the participants. The participants were all married, 77% of them were “breastfed” when grew up and 62% were born through cesarean section. Through repeated analysis, collation, classification, and refinement of the interview results, five core themes related to non-initiation decision-making emerged (Table 2): (1) expected breastfeeding stress; (2) maladjustment to the maternal role; (3) concerns about physiological issues; (4) the lack of knowledge about the risks of artificial feeding; (5) belief that it is better not to initiate than to interrupt.

**Table 1:** Description of mothers(*N *=13)

Participate	Age	Education level	Mode of delivery	Gestational weeks	Postpartum days	Work
**N1**	29	Diploma	Vaginal delivery	38w^+2^	4d	Full-time
**N2**	27	Bachelor’s degree	Caesarean section	39w^+5^	6d	Full-time
**N3**	28	Diploma	Caesarean section	37w^+6^	7d	Full-time
**N4**	24	High school	Caesarean section	40w^+1^	6d	No work
**N5**	23	High school	Vaginal delivery	39w ^+3^	3d	Full-time
**N6**	29	Bachelor’s degree	Caesarean section	38w ^+5^	6d	Full-time
**N7**	28	Diploma	Vaginal delivery	39w ^+5^	4d	Part-time
**N8**	36	High school	Caesarean section	39w ^+1^	5d	Full-time
**N9**	27	Diploma	Vaginal delivery	40w ^+3^	3d	Full-time
**N10**	30	Diploma	Vaginal delivery	39w ^+4^	4d	Full-time
**N11**	31	Master’s degree	Caesarean section	39w ^+3^	5d	Full-time
**N12**	27	Diploma	Caesarean section	38w ^+5^	6d	Full-time
**N13**	23	High school	Caesarean section	39w ^+2^	5d	No work

**Table 2:** Data analysis structure

Theme	Theme Definition	Categories	Categories Definition
Expected breastfeeding stress	This theme describes the difficulties women expect to face when they decide for breastfeeding	1.Breastfeeding difficulties 2.Conflict with family 3.Conflict with work	1.Insufficient milk supply, cracked nipples, pain, and sucking problems 2.Doubts, judgments and compulsion from family 3.Inadequate maternity leave affects breastfeeding
Maladjustment to the maternal role	This theme describes non-initiation as a sign of maladjustment to the maternal role	1.Unwillingness to be a mother 2.Return to the life before motherhood	1.Lack of willingness to be a mother 2.Eager to return to childless life
Concerns about physiological issues	This theme describes how physiological problems affect the decision-making in breastfeeding	1.Fear to transmit infections 2.No guarantee of nutrition 3.Poor condition of the breast	1.Fear to infect children with their own diseases 2.Mothers are worried that they cannot provide adequate nutrition for their children 3. Flat breasts and inverted nipples
The lack of knowledge about the risks of artificial feeding	This theme describes mothers’ understanding of formula milk as a good substitute for human milk	1.Belief that feeding formula milk is more convenient 2.Lack of information about the shortcomings of formula milk	1.Mothers believe that formula feeding is easier than breastfeeding 2.Lack of correct information about formula milk and its potential risks
Belief that it is better not to initiate than to interrupt	This theme describes the subtle psychology of the decision-making for the non-initiation of breastfeeding: mothers believe that it is better, for both mothers and babies, not to initiate breastfeeding than to early interrupt breastfeeding	1.Troubles due to early interruption 2.Non-initiation is good for the mother	1.Mothers believe that early interruption may lead to rejection of milk powder and indigestion 2.Mothers believe that non-initiation can make them rest and recover better

### Expected breastfeeding stress

The “expected breastfeeding stress” was the major theme extracted from the data of this study, and “stress” was the most frequently mentioned word among the participants. Many participants expressed their worries about breastfeeding problems, including lack of milk, fatigue, chapped nipples, and pain, etc. They expected to feed their babies easily and comfortably, but they knew few successful breastfeeding cases in real life and many cases of early weaning, which made them believe that easy and comfortable breastfeeding was almost impossible.

*N4: Breastfeeding is too difficult, especially the lack of mother’s milk is common. They (breastfeeding mothers) have to desperately stimulate milk production.*

*N11: I have a colleague who breast-fed her baby with mastitis three times. It turned out she had to finally cut her breast open, and an open wound was left on her breast for a long time to expel the pus. It was so terrible.*

Mother’s milk is a natural connection between mother and child, and it is also the whole source of nutrition for the infants at the first 6 months. Participants expressed that people usually believe that the quality and quantity of mother’s milk directly determine the health status of the baby. Mothers have to face the scrutiny of others when raising the baby. When the baby shows an undesired behavior (crying or refusing to sleep) or developmental problems (baby is overweight or underweight), the quality or quantity of the mother’s milk is considered as the reason. Consequently, mothers have to face multiple stressors, like frustration, family conflicts, taboos during lactation, and special dietary requirements. They describe their feeling as if they were fighting alone.

*N2: One of my girlfriends was driven crazy by her mother-in-law because the older generation believe that the body growth and development are attributed to the quality and volume of mother’s milk. She was forced to drink pig trotter soup every day. Her mother-in-law was always nagging other children were chubby while their child was very thin. Child diarrhea was also blamed on her diet. It was a great stress for her.*

Not all mothers have enough time and energy to take care of their children as a full-time job. After returning to work, continuing to breastfeed presents a big difficulty. A number of participants have to return to work within 8–20 weeks after delivery, which was the most important factor for refusing breastfeeding. They worried the lack of time and energy due to their work, which would seriously affect the effect of breastfeeding and the development of the baby. They were also worried that breastfeeding will affect the results of their work.

*N1: I have to go to work, and I cannot breastfeed. If I cannot sleep well at night, my work will be greatly affected. My home is an hour away from my workplace, and I cannot return for breastfeeding. In addition, I often have to go on business trips, so for me it is better to feed formula milk directly.*

*N9: I heard that bottles and formula are very difficult to be accepted by babies who are used to mother’s milk. I am sure that I will not have enough time to feed my baby, and he will be miserable when I go to work.*

### Maladjustment to the maternal role

“Maladjustment to the maternal role” was an important theme emerged from participants’ experiences, though only a few participants mentioned it. The process of pregnancy and childbirth is a great stress for every woman, and growing into the role of a new mother who has to take care of a baby is a great change for every woman. During this adaptation, failure to debug successfully will lead to various psychological and behavioral changes, resulting in the maladjustment to the maternal role.

Being influenced by traditional Chinese culture, breasts are regarded as a symbol of sex. Participants hoped that they could still remain mysterious and energetic after giving birth, which was incompatible with breastfeeding in their opinion. One of the participants showed a strong sense of resistance to breastfeeding, and she once thought about pumping milk to feed the infant and avoid physical touch, but she gave it up after giving birth.

*N12: I cannot accept breastfeeding because I cannot stand the baby touching my breast. It makes me feel disgusted, and it seems that mothers are not themselves any longer once they become mothers. Especially breastfeeding in public is so embarrassing.*

In the course of the interview, many participants expressed their nostalgia for the independent life before pregnancy. They did not want to become a mother at all, hoping to return to their previous life quickly. The inadaptability to the maternal role made them reject all maternal behaviors, including breastfeeding.

*N6: I did not want this baby at all, but my family felt I was old enough to have children. I made a deal with my family at the very beginning: I would be only responsible for giving birth, and they would take care of everything else.*

*N3: I am not happy to be a mother. I just feel that I have completed a task. Also, milk powder feeding is good and does not affect my normal life.*

### Concerns about physiological issues

“Concerns about physiological issues” was one of the strongest themes emerged from the participants’ interviews. Physiological problems were also one of the reasons why mothers refused to breastfeed. Although some doctors told them they could breastfeed their babies, mothers were afraid to try and they gave up breastfeeding to avoid the possible trouble it may cause. Although inverted nipples are very common and do not necessarily affect breastfeeding, two participants refused to start breastfeeding because they thought they would not be able to breastfeed smoothly.

*N10: My nipples are* inverted *sunken, which may cause trouble for the atresia. I know some people believe that it does not matter, but I am still very scared. I do not want to wait until the nipple breaks to decide to wean.*

Other two participants who were chronically ill and had regular antenatal examinations during pregnancy were able to recognize the advantages of breastfeeding for the child’s development. However, they were worried that breastfeeding would affect the children’s health and the children may even develop the mothers’ diseases. Moreover, they were worried about whether their milk can meet the needs of infant growth and development. Therefore, they believed artificial feeding is more suitable than breastfeeding.

*N8: I always thought that patients with hepatitis B cannot get pregnant. I would not have waited until the age of 36 to have a baby if I had known that hepatitis B patients can get pregnant. Breastfeeding is the best choice, but it is not suitable for me because I do not want to infect my baby. I do not want to take any risk that may affect my baby’s health.*

*N7: I have always been anemic. Before I got pregnant, I asked the doctor if anemia would lead to insufficient milk. I thought about breastfeeding for a long time and finally decided against it. Even if I had enough human milk, its nutritious value may be lower than that of other mothers, and the growth and development of the baby may be affected. Artificial feeding is better for both myself and my baby.*

### The lack of knowledge about the risks of artificial feeding

 “The lack of knowledge about the risks of artificial feeding” was a major theme emerged from the participants’ interviews. Participants generally expressed that they considered mother’s milk as the best feeding option, but they also believed that formula milk is good enough to meet the nutritional needs of babies and even better than mother’s milk in some aspects. None of the participants was informed about the risks that maybe caused by artificial feeding, for example, obesity and increased incidence of otitis media in adulthood. Some participants directly said that they did not see any disadvantages of formula and believed that artificially fed babies were equally healthy or even better developed in height, weight, and other indicators than breast-fed babies.

*N6: I do not think that formula has any disadvantages. Artificially fed babies are usually healthier and taller than breast-fed babies.*

*N13: I grew up on formula. At that time, the quality of milk powder was not as good as that of today’s formula milk. The mothers who breast-fed around me actually envied the mothers who fed formula for the stronger babies.*

### Belief that it is better to not initiate than to interrupt

 Almost every participant mentioned this topic “It is better to not initiate than to interrupt” unexpectedly. Although participants expressed different reasons for refusing breastfeeding, most of them preferred to not initiate than to interrupt. They believed that interruption of breastfeeding at an early stage may lead to the rejection of formula milk, which implies that they considered formula milk as a necessity for infants. Furthermore, the participants mentioned that weaning will lead to indigestion and crying, and they were worried that this may affect the development of the infants. The participants expected that children who directly received formula at birth will grow up smoothly without troubles caused by the interruption of breastfeeding.

*N5: If the babies are breastfed for only two or three months, the mother and the baby may have to go through a separation even though they were just getting used to breastfeeding. Conversion from human milk to formula milk usually causes indigestion, and the baby’s development may slow down. I think this conversion causes some kind of hurt, and I would rather choose formula directly.*

*N12: It is better not to start, since it causes more troubles (the baby’s rejection of formula milk and crying caused by weaning) than benefit to breastfeed only for a few months.*

## Discussion

Although mothers generally understand the protective effects of breastfeeding on mothers and children, the awareness of “breastfeeding is best” message does not necessarily equate to breastfeeding continuation beyond the immediate postpartum period [29]. Therefore, we conducted a semi-structured interview in Jingzhou City (China) with postpartum mothers who did not breastfeed after giving childbirth and analyzed the reasons in order to understand the mothers’ views on non-initiation of breastfeeding. To the best of our knowledge, this is the first qualitative study on non-initiation. In this study, the decision-making was a complex and multifaceted process and more nursing assistance needs to be provided.

According to the framework recommended by the Lancet series [6], the structural context for breastfeeding in this study was not supportive enough. Pregnant mothers would like to communicate with new mothers or other elder mothers in order to gain more parenting experience and they may receive negative cognition of breastfeeding such as breastfeeding difficulties、conflict with family or work and traditional customs [30–32]. These problems can cause great psychological stress to mothers, which is closely related to their breastfeeding behavior. Inadequate maternity protection policies might be crucial. Although the International Labour Organization recommends that maternity leave must be at least 18 weeks [33], China still neither meets this requirement nor the demand for six months of exclusive breastfeeding as per WHO recommendations [2]. Furthermore, given the lack of paternity leave, the first-time mothers have to take care of their children by themselves alone or with parents or in-laws, causing the mothers more stress.

The aggressive marketing of the products of infant artificial milk manufacturers over the past decades has been highly successful in creating a social preference for infant artificial milk over breastfeeding [34], whereby artificial milk feeding is perceived as the normal infant feeding method [35]. The unethical marketing of commercial milk formula is playing a crucial role; millions of dollars are invested in such marketing campaigns for a long time, undermining breastfeeding in China. As a result, though most Chinese mothers perceive breastfeeding as the optimal feeding method, a substantial proportion of them consider bottle feeding to be more convenient, less tiring, and more nutritious than breastfeeding [36]. Due to social stress, Chinese parents’ unique anxiety and insecurity [37] make them want to raise more “high-quality” children. As formula feeding often cause babies taller and fatter in appearance, it corresponds better to the objectives of Chinese parents.

A large proportion of first-time mothers gave birth by cesarean section in this study. China is among the countries with the highest cesarean section rates in the world, and 40% of cesarean sections were performed without medical indication [38]. Furthermore, though the Early Essential Newborn Care (EENC) has been reported to be significantly associated with the initiation of early breastfeeding [39], the current childbirth and early newborn care policies and practices in China are not aligned with the WHO recommendations for some major interventions [40]. In addition, the medical advice to give commercial milk formula is not rare in China. These inappropriate medical interventions might affect the decision of non-initiating breastfeeding potentially.

Consistent with previous study by Horwood et al. [19], physical factors were reported in this study. Mothers were worried that infectious diseases, chronic diseases, and breast abnormalities may negatively affect breastfeeding, and they believed that these problems would lead to more breastfeeding stress. Obviously, mothers have not gained enough knowledge and confidence to practice breastfeeding in these cases, though a number of studies [41, 42] have confirmed that breastfeeding is compatible with such diseases and abnormalities. This situation might be related to the doctor’s fear of responsibility. The patient-physician relationship is facing a trust crisis in China [43]. Although these physical problems do not affect breastfeeding and mothers’ or infants’ health, the doctors do not stick to the recommendations, just in case.

At the personal level, affected by the cost of education, housing prices, and other factors, the fertility intention of Chinese mothers declined in recent years. Baumgartner et al. [44] reported that exclusive artificial feeding with bottles was more likely to be chosen when the pregnancy was unplanned. Consistent with this study, new mothers who got pregnant involuntarily expressed resistance to breastfeeding in the present study. As we know, maternal psychological aspects were associated with the breastfeeding pattern [45]. The adaptation to the role of motherhood was found to be an important factor affecting the decision-making of the feeding method. Therefore, future research should pay more attention to the identification of motherhood, and it is very important to evaluate the level of adaptation to the maternal role and the risk factors for maladjustment.

In addition, mothers unexpectedly reported that non-breastfeeding is better than short-term breastfeeding. Though short-term breastfeeding can be very helpful and even small amounts of mother’s milk strongly influence the accumulation of viral populations in the infant gut and provide a protective effect against potentially pathogenic viruses [46], which has not been understood by the mothers. They reported that interruption of breastfeeding would cause troubles for themselves and their babies. As a result, they directly preferred artificial feeding even though short-term breastfeeding would have been possible. This decision-making indicates that there are still some deficiencies in the current breastfeeding education in China.

Previous breastfeeding promotion strategies were mainly aimed at the protective effects and methods of breastfeeding. In this study, we found that the mothers were very clear about the protective effects of breastfeeding, but lack of knowledge about the disadvantages and risks of artificial feeding still led to the rejection of breastfeeding. The current breastfeeding education does not meet the needs of mothers in China. Moreover, previous study [47] found that the attitudes of relatives also affect the self-efficacy and behavior of Chinese mothers in breastfeeding. In the future, the promotion of breastfeeding also needs to focus on the possible risks of formula feeding, and the promotion should not only target pregnant mothers and new mothers, but also all populations including their families.

The findings in this study have practical implications and call for the implementation of new policies to improve and build breastfeeding-friendly families and hospitals. Expanding the awareness of breastfeeding in the population and enhancing the pertinence of breastfeeding awareness are very important. In addition, restricting the marketing of formula milk in China might also be a matter of concern.

This study was conducted in the hospitals of a medium-sized city. The results might be affected by the local culture and might not be popularized in other regions. The mothers were interviewed in the maternity ward rather than after discharging from the hospital, and in the context of very high initiation rates in the hospitals, these factors might have affected the participants’ views toward breastfeeding and they might not have answered the questions as freely as they wanted. The researcher’s personal experience with successful long-term breastfeeding might have an influence on the analysis of this study. Despite the limitations, the present study provides a meaningful contribution to the breastfeeding literature and offers novel information on the reasons for the non-initiation of breastfeeding among a sample of first-time mothers in China.

## Conclusions

The study findings contribute to our understanding of how mothers make the decision of non-initiation and several barriers to breastfeeding initiation in China that warrant addressing. The environment for mothers to breastfeed is not supportive enough, the medical staff and families should be held responsible for the non-initiation of breastfeeding. Therefore, more professionals are needed to support mothers to solve their problems and breastfeeding education should be further expanded. The findings in this study may assist health policy makers to develop intervention programs.

## Data Availability

The datasets generated during the current study are not publicly available due to confidentiality of the participants, but are available from the corresponding author on reasonable request.

## References

[1] Walters DD, Phan LTH, Mathisen R (2019). The cost of not breastfeeding: global results from a new tool. Health Policy Plan.

[2] World Health Organization (2003). UNICEF. Global Strategy for Infant and Young Child Feeding.

[3] Yang Z, Lai J, Yu D, Duan Y, Pang X, Jiang S (2016). Breastfeeding rates in China: a cross-sectional survey and estimate of benefits of improvement. Lancet.

[4] UBIC Consulting. Ingredients used in the world infant formula market. 2020. https://ubic-consulting.com/reports/nutrition/ingredients-for-the-world-infant-market-report/

[5] China Development Reaserch Foundation. Investigation report on Influencing Factors of breastfeeding in China.2019. https://cdrf.org.cn/jjh/pdf/mu.pdf

[6] Rollins NC, Bhandari N, Hajeebhoy N, Horton S, Lutter CK, Martines JC (2016). Why invest, and what it will take to improve breastfeeding practices?. Lancet.

[7] Thulier D, Mercer J (2009). Variables associated with breastfeeding duration. Journal of Obstetric, Gynecol Neonat Nur.

[8] Morrison AH, Gentry R, Anderson J (2019). Mothers’ reasons for early breastfeeding cessation. MCN-Am J Matern-Chil.

[9] Napierala M, Mazela J, Merritt TA, Florek E (2016). Tobacco smoking and breastfeeding: effect on the lactation process, breast milk composition and infant development. A critical review. Environ Res.

[10] Nomura K, Minamizono S, Nagashima K, Ono M, Kitano N (2020). Maternal Body Mass Index and Breastfeeding Non-Initiation and Cessation: A Quantitative Review of the Literature. Nutrients.

[11] Schliep KC, Denhalter D, Gren LH, Panushka KA, Singh TP, Varner MW (2019). Factors in the hospital experience associated with postpartum breastfeeding success. Breastfeed Med.

[12] Steurer LM (2017). Maternity leave length and workplace policies’ impact on the sustainment of breastfeeding: Global perspectives. Public Health Nurs.

[13] Allen JA, Li R, Scanlon KS, Perrine CG, Chen J, Odom E (2013). Progress in increasing breastfeeding and reducing racial/ethnic differences—United States, 2000–2008 births. Mmwr-Morbid Mortal W.

[14] Centers for Disease Control and Prevention (CDC). Breastfeeding report card: Progressing toward national breastfeeding goals. Atlanta (GA): CDC; 2016. http://www.cdc.gov/breastfeeding/data/report_card.htm

[15] Department of Health. Indicators on Breastfeeding (2013). Quarter 4 2012/13.

[16] Xu F, Qiu L, Binns CW, Liu X (2009). Breastfeeding in China: a review. Int Breastfeed J.

[17] Ogbuanu CA, Probst J, Laditka SB, Liu J, Baek J, Glover S (2009). Reasons why mothers do not initiate breastfeeding: a southeastern state study. Mothers’s Health Issues.

[18] Hall H, McLelland G, Gilmour C, Cant R (2014). ‘It’s those first few weeks’: Mothers’s views about breastfeeding support in an Australian outer metropolitan region. Mothers Birth.

[19] Horwood C, Haskins L, Engebretsen I, Connolly C, Coutsoudis A, Spies L (2020). Are we doing enough? Improved breastfeeding practices at 14 weeks but challenges of non-initiation and early cessation of breastfeeding remain: findings of two consecutive cross-sectional surveys in KwaZulu-Natal, South Africa. BMC Public Health.

[20] Hofvander Y (2003). Why mothers don’t breastfeed: a national survey. Acta Paediatrica.

[21] Hesse-Biber SN, Leavy P. The practice of qualitative research. 2 ed: Sage Publications; 2010. https://books.google.com.hk/books?id=FVjcZk7mCFwC&lpg=PR1&ots=6vRtpefgd6&dq=The%20practice%20of%20qualitative%20research&lr&hl=zh-CN&pg=PR3#v=onepage&q=The%20practice%20of%20qualitative%20research&f=false

[22] Patton MQ. Qualitative evaluation methods: Sage publications Beverly Hills, CA; 1980. https://www.ojp.gov/ncjrs/virtual-library/abstracts/qualitative-evaluation-methods

[23] Yang X, Ip WY, Gao LL (2018). Maternal intention to exclusively breast feed among mainland Chinese mothers: A cross-sectional study. Midwifery.

[24] Mason M (2010). editor Sample size and saturation in PhD studies using qualitative interviews.

[25] Kallio H, Pietilä AM, Johnson M, Kangasniemi M (2016). Systematic methodological review: developing a framework for a qualitative semi-structured interview guide. J Adv Nur.

[26] Mayring, P. Qualitative Content Analysis. In: U. Flick, E. von Kardoff and I. Steinke (Eds). A Companion to Qualitative Research. Glasgow: SAGE; 2004. p. 159–76.

[27] Hsieh H-F, Shannon SE (2005). Three approaches to qualitative content analysis. Qual Health Res.

[28] Creswell JW, Creswell JD (2018). Research Design: Qualitative, Quantitative, and Mixed Methods Approaches.

[29] Debevec AD, Evanson TA (2016). Improving breastfeeding support by understanding mothers’s perspectives and emotional experiences of breastfeeding. Nur Mothers’s Health.

[30] Andrews T, Knaak S (2013). Medicalized mothering: Experiences with breastfeeding in Canada and Norway. Sociol Rev.

[31] Kuswara K, Knight T, Campbell KJ, Hesketh KD, Zheng M, Bolton KA (2021). Breastfeeding and emerging motherhood identity: an interpretative phenomenological analysis of first time Chinese Australian mothers’ breastfeeding experiences. Mothers Birth.

[32] Tang L, Zhu R, Zhang X (2016). Postpartum depression and social support in China: a cultural perspective. J Health Commun..

[33] International Labour Organization (ILO). International Labour Standards on Maternity Protection. 2015 [cited 2015 Jun 22]. Available from: http://www.ilo.org/global/standards/subjects-covered-by-international-labour-standards/maternity-protection/lang%2D-en/index.htm.

[34] Kaplan DL, Graff KM (2008). Marketing breastfeeding—reversing corporate influence on infant feeding practices. J Urban Health.

[35] Bartick MC, Walker M, Bagley D, Wiessinger D, editors. Breastfeeding as normal: An urban breastfeeding advertising campaign inspired by the tobacco industry. American Public Health Association’s 134th Annual Meeting-Public Health and Human Rights. Boston, MA: APHA; 2006.

[36] Zhang K, Tang L, Wang H, Qiu L-Q, Binns CW, Lee AH (2015). Why do mothers of young infants choose to formula feed in China? Perceptions of mothers and hospital staff. Int J Environ Res Public Health.

[37] Kuan T (2015). Love’s Uncertainty: The politics and ethics of child rearing in contemporary China.

[38] Deng W, Klemetti R, Long Q, Wu Z, Duan C, Zhang WH (2014). Cesarean section in shanghai: women’s or healthcare provider’s preferences?. BMC Pregnancy Childbirth.

[39] Wang CR, Li XY, Zhang L, Wu LM, Tan L, Yuan F (2020). Early essential newborn care is associated with increased breastfeeding: a quasi-experimental study from Sichuan Province of western China. Int Breastfeed J.

[40] Xu T, Yue Q, Wang Y, Murray J, Sobel H (2018). Childbirth and Early Newborn Care Practices in 4 Provinces in China: A Comparison with WHO Recommendations. Glob Health Sci Pract.

[41] World Health Organization (2016). Guideline: Iron Supplementation in Postpartum Mothers.

[42] Zhang L, Gui X, Fan J, Wang B, Ji H, Yisilafu R (2014). Breast feeding and immunoprophylaxis efficacy of mother-to-child transmission of hepatitis B virus. J Matern-Fetal Neo M.

[43] Huang RC, Song XT, Zhang DF, Xu JY, Montori VM (2019). Preferences and attitudes of young Chinese clinicians about using a shared decision making tools for communicating cardiovascular risk. Chronic Dis Transl Med.

[44] Baumgartner T, Bhamidipalli SS, Guise D, Daggy J, Parker CB, Westermann M (2020). Psychosocial and Sociodemographic Contributors to Breastfeeding Intention in First-Time Mothers. Matern Child Health J.

[45] Gila-Díaz A, Carrillo GH, López de Pablo ÁL, Arribas SM, Ramiro-Cortijo D (2020). Association between maternal postpartum depression, stress, optimism, and breastfeeding pattern in the first six months. Int J Environ Res Public Health.

[46] Liang G, Zhao C, Zhang H, Mattei L, Sherrill-Mix S, Bittinger K (2020). The stepwise assembly of the neonatal virome is modulated by breastfeeding. Nature.

[47] Bai DL, Fong DYT, Lok KYW, Tarrant M (2016). Relationship between the infant feeding preferences of Chinese mothers’ immediate social network and early breastfeeding cessation. J Hum Lact.

